# Tea, Coffee and Liquors

**Published:** 1870-12

**Authors:** 


### TEA, COFFEE AND LIQUORS.

All are stimulants, and within ten minutes after taking them we lose the sense of fatigue which depressed us, and the feeling of hunger which seemed to be gnawing at our vitals. By using them largely, we are able to work until the very moment that paralysis, apoplexy, or death, shows how much the system has been outraged.

The action of the stimulus is to force the blood to the various parts of the body, when it would not have gone there sufficiently by its natural action. This blood contains nutriment—hence the feeling of hunger abates—but a mere stimulant having no nutriment, the time comes when the blood has yielded up all it has, and the body dies of starvation, and debility, and utter exhaustion.

Persons who are starved to death become idiotic towards the last, because there is not nourishment in the blood to feed the brain, to keep up its activities.

It was observed during the potatoe famine in Ireland, that when money, instead of food, was given to the starving, they uniformly spent it in tea, tobacco, or spirits, because they found that they removed the sense of debility and hunger at a cheaper rate than solid food, and the relief was more instantaneous; but they failed to notice that it was also more transient, and thus more deceptive; they gave no actual strength to the body—they only seemed to do it; they merely brought the very last reserves "to the front," but added not a man to the general army; so when the last reserves were used up, the battle of life was over, and death was victorious. Hence, strength, kept up by the use of mere stimulants, is always at the risk of reason and life. True strength, real recuperation, comes from the digestion of nutritious food, and can come from no other source.

